# Digitale Hebammenbetreuung in der COVID-19-Pandemie in Deutschland – Akzeptanz bei Müttern

**DOI:** 10.1007/s00103-023-03666-8

**Published:** 2023-02-10

**Authors:** Nicola H. Bauer, Dagmar Hertle, Luisa Schumacher

**Affiliations:** 1 Institut für Hebammenwissenschaft, Medizinische Fakultät, Universität zu Köln, Kerpener Str. 34, 50931 Köln, Deutschland; 2 BARMER Institut für Gesundheitssystemforschung, Wuppertal, Deutschland; 3 Bereich Forschung/Beratung, Deutsches Krankenhausinstitut, Düsseldorf, Deutschland

**Keywords:** Digitalisierung, Hebammenbetreuung, Covid-19-Pandemie, Evaluation, Mütter, Digitalization, Midwifery care, Covid-19 pandemic, Evaluation, Mothers

## Abstract

**Hintergrund:**

Zur Sicherstellung der ambulanten Hebammenversorgung während der COVID-19-Pandemie wurden im März 2020 in Deutschland erstmals digitale Hebammenleistungen ermöglicht. Ziel der Studie „Digitale Hebammenbetreuung im Kontext der Covid-19-Pandemie“ war eine erste Evaluation der neu eingeführten digitalen Angebote aus Sicht von Hebammen und Müttern. In dieser Publikation werden die Ergebnisse der Mütterbefragung dargestellt.

**Methode:**

Im Februar und März 2021 wurde die Querschnittstudie durchgeführt. Es wurden bei der BARMER versicherte Frauen, die zwischen Mai und November 2020 ein gesundes Kind geboren haben, bundesweit mithilfe eines explorativ entwickelten Online-Fragebogens zu Inanspruchnahme, Zufriedenheit und den Potenzialen der digitalen Hebammenbetreuung in Schwangerschaft und Wochenbett anonymisiert befragt.

**Ergebnisse:**

1821 Mütter nahmen an der Befragung teil. Rund ein Drittel der antwortenden Frauen hatte in der Schwangerschaft und/oder im Wochenbett digitale Hebammenleistungen in Anspruch genommen und diese Leistungen zu über 80 % positiv bewertet. Aus Sicht der Befragten eignen sich Kurse und Beratung sehr gut, wohingegen die Wochenbettbetreuung oft die Präsenz der Hebamme erfordere. Als Vorteile wurden der Infektionsschutz sowie die Zeit- und Wegeersparnis gesehen.

**Fazit:**

Die COVID-19-Pandemie hat auch in der Hebammenversorgung einen Digitalisierungsschub bewirkt. Die digitalen Angebote wurden von den freiberuflichen Hebammen schnell umgesetzt. Diese wurden von den Frauen gut angenommen und können die Betreuung in Präsenz sinnvoll ergänzen. Chancen und Weiterentwicklungsmöglichkeiten der digitalen Hebammenbetreuung sollten nun genutzt werden.

## Abstract (en)

**Background:**

To ensure outpatient midwifery care during the COVID-19 pandemic, digital midwifery services were enabled for the first time in Germany in March 2020. The aim of the survey “Digital midwifery care in the context of the Covid-19 pandemic” was to conduct an initial evaluation of the newly introduced digital services from the perspective of the midwives and the users. This publication presents the results of the mothers’ survey.

**Method:**

In February and March 2021, a cross-sectional study with an online survey was conducted. Women insured with BARMER who gave birth to a healthy child between May and November 2020 were surveyed anonymously throughout Germany using an exploratively developed online questionnaire on the utilization, satisfaction, and potential of digital midwifery care in pregnancy and the postpartum period.

**Results:**

Feedback was provided by 1821 mothers. Around one third of the responding women had used digital midwifery services during pregnancy and/or the postpartum period and rated these services positively by over 80%. From the respondents’ point of view, courses and counselling are very well suited whereas postpartum care often requires the midwife’s presence. Advantages were seen in infection control and in saving time and travel.

**Conclusion:**

The COVID-19 pandemic has become a catalyst for digitalization in midwifery care. The digital services were quickly implemented by freelance midwives and well accepted by women and can usefully complement the care provided in the presence of the midwife. The opportunity to utilize and further develop these offers should be taken.

## Hintergrund

Im Jahr 2020 wurden in Deutschland 773.144 Kinder geboren [1]. Frauen können mit Beginn der Schwangerschaft, während der Geburt und in den ersten 9 Monaten nach der Geburt bzw. bis zum Ende der Stillzeit die Betreuung durch freiberuflich tätige Hebammen im ambulanten Sektor in Anspruch nehmen [2, 3].

Zu deren Aufgabenspektrum zählen u. a. die Durchführung von Vorsorgeuntersuchungen und Beratungen in der Schwangerschaft, Hilfe bei Schwangerschaftsbeschwerden, Durchführung von Geburtsvorbereitungskursen, Betreuung bei Fehlgeburten, Betreuung und Überwachung des Geburtsverlaufs und Durchführung eventuell notwendiger Notfallmaßnahmen sowie die Betreuung des Wochenbetts und der Stillzeit [2, 3]. Das Modell der aufsuchenden Wochenbettbetreuung über einen Zeitraum von 12 Wochen ist weltweit einzigartig [4].

Hebammen sind eigenverantwortlich tätig und die Betreuung erfolgt nach dem biopsychosozialen Modell. Die Lebensphase von Schwangerschaft, Geburt und Wochenbett ist von besonderer Bedeutung, da die dabei gemachten Erfahrungen kurz-, mittel- und langfristige Auswirkungen auf die physische und psychische Gesundheit von Mutter, Kind und Familie haben können [5].

### COVID-19-Pandemie und digitale Hebammenleistungen

Im Kontext der COVID-19-Pandemie hat die Digitalisierung der gesundheitlichen Versorgung einen höheren Stellenwert erfahren und die pragmatische Umsetzung neuer Versorgungskonzepte hat stark zugenommen [6].

Bis zum Beginn der COVID-19-Pandemie im Jahr 2020 sah der Vertrag über die Versorgung mit Hebammenhilfe nach § 134a Fünftes Buch Sozialgesetzbuch (SGB V) in § 6 Abs. 1 [7] vor, dass freiberuflich tätige Hebammen Leistungen persönlich erbringen müssen. Aufgrund der Pandemie dürfen Hebammen seit März 2020 bestimmte Leistungen in der Schwangerschaft sowie im Wochenbett und in der Stillzeit erstmals auch digital anbieten. Die Vereinbarungen des Spitzenverbands der Gesetzlichen Krankenversicherungen (GKV) hatte mit einer befristeten Änderungsvereinbarung [8] die Möglichkeit geschaffen, dass Hebammen Leistungen über alternative Wege erbringen können:

- *Schwangerschaft:* Vorgespräche und Hilfe bei Beschwerden oder bei Wehen per Telefon oder per Videotelefonie und Geburtsvorbereitungskurse per Live-Videotelefonie;
- *Wochenbett:* Wochenbettbetreuung online entweder per Telefon oder vorrangig per Live-Videotelefonie, Rückbildungskurse per Live-Videotelefonie, Beratung bei Still- und Ernährungsschwierigkeiten, wenn sie mindestens 20 min dauern.

Das Digitale-Versorgung-und-Pflege-Modernisierungs-Gesetz [9] erlaubt seit Juni 2021 eine Verstetigung dieser digitalen Angebote, sodass nun eine dauerhafte Leistungserbringung der Hebammenbetreuung in der Schwangerschaft und im Wochenbett möglich ist.

### Auswirkungen der COVID-19-Pandemie

Durch die COVID-19-Pandemie haben sich das Leben und die gesundheitliche Versorgungsituation weltweit verändert. Die umgesetzten Maßnahmen zwischen März 2020 und Mai 2021 und deren Auswirkungen unterschieden sich je nach Bundesland, Region und 7‑Tage-Inzidenzen [10, 11]. Insbesondere in den ersten Monaten der Pandemie herrschte eine große Unsicherheit darüber, wie stark sich eine SARS-CoV-2-Infektion auf Schwangere und ihre Ungeborenen auswirkt und welche Folgen diese haben kann.

Schwangere, Gebärende und Wöchnerinnen sind als vulnerable Gruppe anzusehen, die aufgrund der COVID-19-Pandemie und den damit verbundenen Regelungen und Schutzmaßnahmen in ihrem physischen und psychischen Wohlbefinden sowie im Zugang zu gesundheitlichen Angeboten eingeschränkt wurden. So untersagten z. B. manche Geburtskliniken zeitweise eine Geburtsbegleitung durch Partner*in oder Begleitperson. Zu Beginn der Pandemie war zunächst unklar, ob und in welcher Form die ambulante Hebammenbetreuung weiterhin angeboten werden kann. Zusatzbelastungen entstanden durch fehlende Betreuungsmöglichkeiten für weitere Kinder. Insbesondere Frauen mit psychischen Belastungen oder vorangegangenen negativen oder traumatischen Geburtserlebnissen waren zum Teil stark davon betroffen [12, 13]. Für marginalisierte Gruppen, wie z. B. geflüchtete Frauen, war die Lage besonders schwierig, da die Inanspruchnahme von telefonischen oder digitalen Angeboten aufgrund der Sprachbarrieren meist nicht möglich war und sie eine aufsuchende Betreuung nicht wahrnehmen konnten. Es kam zu Versorgungsschwierigkeiten, so dass Frauen mit Risikoschwangerschaften nicht ausreichend betreut wurden oder eine adäquate Betreuung durch Besuchseinschränkungen in Kliniken und fehlende Sprachmittlung durch eine dritte Person nicht gewährleistet werden konnte [14].

In der britischen „COVID-19 New Mum Study“ wurden 1329 Frauen mit einem Kind im Alter von bis zu 12 Monaten zu ihren Erfahrungen während des Lockdowns befragt. Die Teilnehmerinnen gaben an, sich niedergeschlagen (56 %) und einsam (59 %) zu fühlen sowie reizbar (62 %) und besorgt (71 %) zu sein [15]. Im Mai und Juni 2020 wurden 2840 Frauen in Australien zu ihren Erfahrungen mit der Betreuung während der Schwangerschaft und der Geburt befragt. Circa ein Drittel der Befragten war unzufrieden mit ihrer Betreuung. Am häufigsten gaben Frauen zudem an, sich ängstlich, unsicher, einsam und isoliert gefühlt zu haben. Auch Studien aus der Türkei [16], Irland [17] und Kanada [18] berichten von erhöhten Werten an Angst und depressiven Symptomen bei Schwangeren verglichen mit Erhebungen vor der COVID-19-Pandemie.

Eltern (Mütter und Väter) von Säuglingen im Alter von 0 bis 6 Monaten in den Niederlanden berichteten über hochgradigen COVID-19-bedingten Stress (Mütter wiesen im Vergleich zu Vätern ein höheres Stresslevel auf). Zudem zeigten sie während der Pandemie klinisch bedeutsame psychische Gesundheitssymptome (Mütter: 39,7 % Angstzustände, 14,5 % Depressionen; Väter: 37,6 % Angstzustände, 6,4 % Depressionen; [19]). 377 Studienteilnehmerinnen in Deutschland (Schwangere und Wöchnerinnen, die nach dem 13.03.2020 schwanger waren oder bereits geboren hatten) wurden mit einem Fragebogen der internationalen COVGEN-Initiative (COVID generation, https://www.covgen.org/) zu ihren Erfahrungen während der COVID-19-Pandemie befragt. Sie gaben ein signifikant erhöhtes Stresslevel bedingt durch die COVID-19-Pandemie an (*p* < 0,001). Bei 46,9 % der befragten Frauen lag eine Vorerkrankung oder eine schwangerschaftsassoziierte Erkrankung vor. Bei Frauen mit einem Gestationsdiabetes (11,9 %, *n* = 45) war das Stressempfinden – verglichen zu Frauen ohne Vorerkrankungen – durch die COVID-19-Pandemie signifikant erhöht (*p* = 0,006; [20]).

### Digitale Hebammenangebote

Mehrere internationale Studien haben zur Unterstützung von Schwangeren während der COVID-19-Pandemie die Entwicklung von innovativen, digitalen Konzepten zur psychosozialen Betreuung empfohlen. In den Niederlanden wurden für Schwangere sogenannte Online-Communitys entwickelt und über 3 Wochen von einer Psychologin und einer Hebamme angeboten. Die Teilnehmerinnen zeigten nach der Intervention weniger depressive Symptome und Ängste [21]. Schwangere, die während der COVID-19-Pandemie Mitglied einer Online-Unterstützungsgruppe „FaceMums“ im Vereinigten Königreich waren, berichteten, dass sie durch diese Gruppe schneller an Informationen gelangen konnten, da sie hier die Möglichkeit hatten, unkompliziert eine Hebamme online zu kontaktieren. Sehr positiv wurden aber auch die Möglichkeit der Vernetzung und der Kontakt mit anderen Schwangeren gesehen, insbesondere vor dem Hintergrund der Absage von Geburtsvorbereitungskursen [22].

Ein Großteil der freiberuflich tätigen Hebammen hat sich trotz der Herausforderungen seit Beginn der COVID-19-Pandemie recht schnell umgestellt und bietet digitale Hebammenleistungen an [4, 23]. Im Zeitraum zwischen Juli und September 2020 wurden weltweit Hebammen und weiteres Gesundheitspersonal in der geburtshilflichen Versorgung zum Einsatz von Telemedizin aufgrund der COVID-19-Pandemie befragt. Als positiv wurde von den Befragten angesehen, dass die Betreuung trotz der Pandemie ohne Ansteckungsrisiko fortgesetzt werden konnte und zeitsparend ist. Es wurden aber auch Herausforderungen thematisiert: Mangel an Infrastruktur, wenig technische Kenntnisse, finanzielle Hürden – insbesondere für Klient*innen, Sprachbarrieren, Misstrauen und ein erschwerter Zugang für vulnerable Gruppen. Zudem wurde angemerkt, dass bestimmte physische Untersuchungen nicht durchgeführt werden konnten, und ein Großteil der befragten Hebammen gab an, dass ein nonverbales Feedback nicht möglich war [24].

In der Schweiz wurden Hebammen (*n* = 630) zu ihren Erfahrungen mit der „Betreuung auf Distanz“ (Health Care at Distance) im Kontext der COVID-19-Pandemie befragt. 79,5 % (*n* = 501) der befragten Hebammen haben diese angeboten. Am häufigsten wurde die Nutzung eines Telefons (97,6 %, *n* = 491) angegeben, gefolgt von Messengerdiensten (76,1 %, *n* = 382), E‑Mails (43,6 %, *n* = 219), Videotelefonie (54,8 %, *n* = 275) und SMS (66,3 %, *n* = 333). Die digitale Betreuung wurde von 39,5 % der befragten Hebammen als positiv bewertet. Trotz der Vorteile wurde der Nutzen als begrenzt wahrgenommen. Die am häufigsten genannten Nachteile waren Schwierigkeiten beim Erkennen und Bewerten komplexer Situationen sowie das Problem, dass eine große Anzahl von Untersuchungen und Therapien aufgrund der fehlenden physischen Interaktion nicht möglich war. Insbesondere Klient*innen mit psychischen Problemen, Angststörungen, Sprachbarrieren oder geringem technischem Knowhow waren benachteiligt [25].

Im Februar und März 2021 wurde eine Querschnittstudie mit Online-Befragung von freiberuflichen Hebammen und Müttern durchgeführt, die Angebot, Inanspruchnahme, Zufriedenheit und Potenziale der digitalen Hebammenbetreuung in Schwangerschaft und Wochenbett während der COVID-19-Pandemie erfasste (s. Infobox 1). Die gleichzeitige Erhebung der Hebammen- und der Frauenperspektive in der Studie „Digitale Hebammenbetreuung im Kontext der Covid-19-Pandemie“ ist bisher einmalig.

Auch in der Befragung der freiberuflichen Hebammen in der hier beschriebenen Studie werden Chancen und Herausforderungen gesehen. Geäußerte Chancen der digitalen Betreuung sind das verringerte Infektionsrisiko und der höhere Gesundheitsschutz sowie Weg- und Zeitersparnis und flexiblere Arbeitszeiten. Im Hinblick auf die digitale Betreuung sehen Hebammen vor allem die Herausforderung, dass körperliche Untersuchungen digital nicht möglich sind. Weitere Herausforderungen, die angemerkt wurden, sind die aufwendigen Abrechnungsmodalitäten der digitalen Leistungen sowie Fragen und Unsicherheiten bezüglich des Datenschutzes.

Angemerkt wird zudem, dass eine nichtaufsuchende und/oder digitale Wochenbettbetreuung insbesondere in Ballungsgebieten mit einem hohen Bedarf an Hebammenleistungen oder auch einem Hebammenmangel eine sehr gute Möglichkeit darstellt, um mehr Frauen und Familien betreuen zu können. Perspektivisch wünscht sich rund die Hälfte der Hebammen, dass digitale Hebammenbetreuung in der Schwangerschaft (62,7 %, *n* = 972) und im Wochenbett (50,4 %, *n* = 781) auch nach der COVID-19-Pandemie möglich bleibt. Jedoch darf sich die digitale Betreuung aus Perspektive der Hebammen nicht negativ auf die aufsuchende Betreuung in der Schwangerschaft (85,2 %, *n* = 1322) und im Wochenbett (87,4 %, *n* = 1356) auswirken [26, 27].

Aufgrund des weltweit einzigartigen Modells der aufsuchenden Wochenbettbetreuung über 12 Wochen nach der Geburt in Deutschland ist es nur schwer möglich, Studien aus dem internationalen Kontext zu identifizieren, die diese Form der aufsuchenden Wochenbettbetreuung mit der digitalen Wochenbettbetreuung vergleichen.

Da sich die digitale Hebammenbetreuung von der Betreuung in Präsenz unterscheidet, sind das Annahmeverhalten von Frauen bezüglich dieser digitalen Angebote sowie die persönliche Bewertung relevante neue Untersuchungsgegenstände. Daraus leitet sich die Forschungsfrage ab, ob und in welchem Umfang Frauen digitale Hebammenangebote in Anspruch nehmen und welche digitalen Angebote Hebammen machen.

## Methoden

Im Kooperationsprojekt zwischen dem BARMER Institut für Gesundheitssystemforschung (bifg), dem Deutschen Hebammenverband (DHV) und der Hochschule für Gesundheit Bochum (HS Gesundheit) wurde die digitale Hebammenbetreuung im Kontext der COVID-19-Pandemie erstmals mittels einer Befragung von Frauen nach der Geburt und freiberuflich tätigen Hebammen untersucht. Im Zeitraum vom 17.02. bis zum 15.03.2021 wurden Hebammen sowie bei der BARMER versicherte Frauen bundesweit mithilfe zweier explorativ entwickelter quantitativer Online-Fragebögen (Hebammenfragebogen und Versichertenfragebogen)^1^ anonymisiert befragt [26, 28].

Die Konzeption der beiden Fragebögen erfolgte unter Einbezug von Expert*innen aus den Bereichen Fragebogenentwicklung, Versorgungsforschung, Hebammenwissenschaft und Gesundheitswissenschaften sowie aus der Hebammen- und der Mütterperspektive. Am Pretest haben Vertreterinnen der Elterninitiative Mother Hood e. V.^2^ teilgenommen.

Der Versichertenfragebogen umfasste folgende Inhalte: Fragen zur Person und zum Verlauf von Schwangerschaft und Geburt, zur Inanspruchnahme der Hebammenbetreuung in Präsenz und digital, zur eigenen Einstellung zur Technik und technischen Ausstattung sowie zur Annahme und zur Bewertung der digitalen Hebammenbetreuung. Weiterhin interessierte die Sichtweise der befragten Frauen auf Chancen, Herausforderungen und Zukunftsperspektiven der digitalen Hebammenleistungen. Für die jeweils teilnehmende Person wurden nichtrelevante Fragen ausgefiltert.

Es wurden ausschließlich Frauen in die Zufallsstichprobe eingeschlossen (*n* = 18.784), die bei der BARMER versichert waren, zwischen Mai und November des Jahres 2020 ein lebendes Kind zur Welt gebracht und einen Wohnsitz in Deutschland hatten sowie mindestens 18 Jahre alt waren. Die Rekrutierung erfolgte mittels eines postalischen Briefes, in dem über die Befragung und den Datenschutz informiert wurde und um die Teilnahme mit Hilfe eines abgebildeten QR-Codes gebeten wurde. Zusätzlich wurden weitere 350 Frauen via Onlineplattform „Kinderheldin“^3^ über die Befragung informiert und für eine Teilnahme geworben.

Zur Datenerhebung wurde die Plattform Unipark Questback EFS Fall 2020 Releases verwendet, die Datenauswertung erfolgte mit dem Programm IBM SPSS Statistics 27.

## Ergebnisse

Die Stichprobengröße der Befragung umfasste 1821 Frauen aus ganz Deutschland. Hinsichtlich der erhobenen persönlichen Angaben, wie beispielsweise Alter oder Berufs- und Hochschulausbildung, zeigte sich eine recht homogene Zusammensetzung (Tab. 1).

**Table 1:** 

Persönliche Merkmale		Häufigkeit	Prozent (%)	Kumulierte Prozente (%)
*Alter*	18–20 Jahre	1	0,1	0,1
	21–29 Jahre	371	20,4	20,4
	30–39 Jahre	1348	74,0	94,5
	40 Jahre oder älter	101	5,5	100,0
	Gesamt	1821	100,0	–
*Parität*	1 Kind	983	54,0	54,0
	2 Kinder	647	35,5	89,5
	3 Kinder	146	8,0	97,5
	4 Kinder oder mehr	45	2,4	100,0
	Gesamt	1821	100,0	–
*Berufs- und Hochschulausbildung (Mehrfachnennungen)*	Keine Berufsausbildung	42	2,3	–
	Noch in der Berufsausbildung	14	0,8	–
	Noch in der Hochschulausbildung	29	1,6	–
	Abgeschlossene Lehre	960	52,7	–
	Abgeschlossene Fachschule	223	12,2	–
	Universitätsabschluss oder Fachhochschulabschluss	791	43,4	–
	Gesamt	2017	–	–
*Wohnhaft in Deutschland seit …*	… in Deutschland geboren	1721	94,5	94,5
	… weniger als ein Jahr	1	0,1	94,6
	… mehreren Jahren	99	5,4	100,0
	Gesamt	1821	100,0	–
*Muttersprache Deutsch*	Ja	1734	95,2	95,2
	Nein	87	4,8	100,0
	Gesamt	1821	100,0	–
*Der Umgang mit dem Computer, Laptop, Tablet etc. fällt mir leicht*	Trifft gar nicht zu	6	0,3	0,3
	Trifft eher nicht zu	14	0,8	1,1
	Teils, teils	75	4,1	5,2
	Trifft eher zu	328	18,0	23,2
	Trifft voll zu	1398	76,8	100,0
	Gesamt	1821	100,0	–

Die Stichprobe ist nicht im statistischen Sinn repräsentativ für Frauen, die zwischen Mai und November 2020 in Deutschland ein Kind zur Welt gebracht haben. Zum Beispiel waren die Frauen in dieser Stichprobe bei der Geburt ihrer Kinder etwas älter im Vergleich zu Frauen in der Allgemeinbevölkerung Deutschlands, die im selben Zeitraum geboren haben. Zudem haben sie zu einem höheren Prozentsatz einen Universitäts- oder Fachhochschulabschluss (43,4 %) als Frauen in Deutschland allgemein (32,1 %). Es haben vor allem Mütter teilgenommen, die zwischen 30 und 39 Jahre (74,0 %, *n* = 1348) alt waren, im befragungsrelevanten Zeitraum ihr erstes Kind zur Welt gebracht haben (54,0 %, *n* = 983), selbst in Deutschland zur Welt gekommen sind (94,5 %, *n* = 1721) und eine abgeschlossene Lehre (52,7 %, *n* = 960) und/oder einen Universitäts- bzw. Fachhochschulabschluss hatten (43,4 %, *n* = 791). Vor allem Frauen aus Bayern (16,5 %, *n* = 300) und Nordrhein-Westfalen (20,6 %, *n* = 375) haben den Fragebogen ausgefüllt (in Tab. 1 nicht abgebildet). 94,8 % der Frauen (*n* = 2686) gaben an, ihnen falle der Umgang mit dem Computer, Laptop oder Tablet leicht (Tab. 1).

### Annahme der digitalen Hebammenleistungen

**Figure 1:**
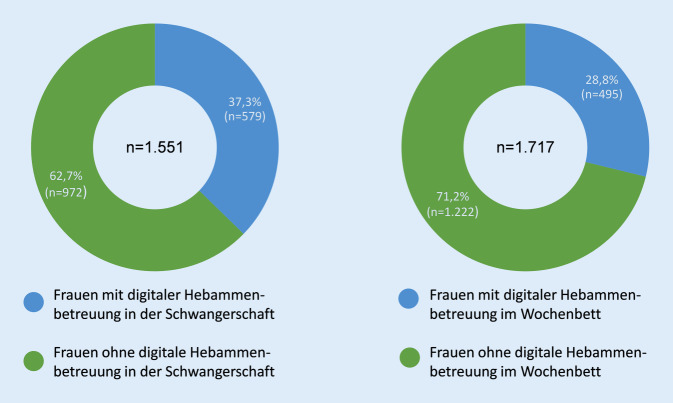

Von den 1821 Müttern haben 1551 Mütter (85,0 %) Hebammenbetreuung in der Schwangerschaft und 1717 Frauen (94,1 %) Betreuung im Wochenbett in Anspruch genommen. Jeweils rund ein Drittel der Frauen wurde in den Lebensphasen (auch) digital durch ihre Hebamme(n) betreut (Abb. 1). Die Frauen hatten die Möglichkeit, unterschiedliche gesetzliche Hebammenleistungen in der Schwangerschaft und im Wochenbett digital und/oder in Präsenz wahrzunehmen.

**Figure 2:**
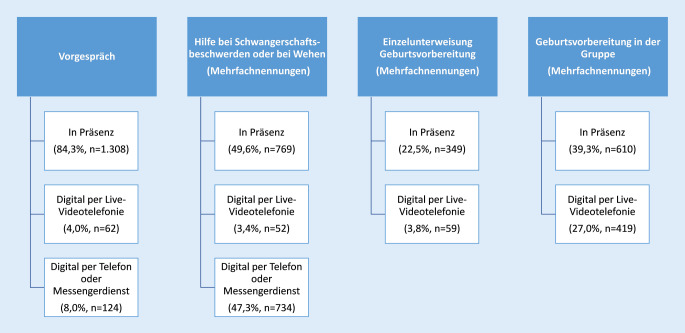

In der Schwangerschaft haben (werdende) Mütter vor allem „Hilfe bei Schwangerschaftsbeschwerden oder bei Wehen“ digital in Anspruch genommen (50,7 %, *n* = 786), jedoch vorrangig per Telefon und nicht per Live-Chat mit Bild und Ton (Abb. 2). Digitale Live-Videotelefonie kam hingegen recht häufig bei der „Geburtsvorbereitung in der Gruppe“ (27,0 %, *n* = 419) zum Einsatz. Das Vorgespräch zwischen werdenden Müttern und Hebammen sowie die Einzelunterweisungen zur Geburtsvorbereitung wurden hingegen vorrangig in Präsenz wahrgenommen.

**Figure 3:**
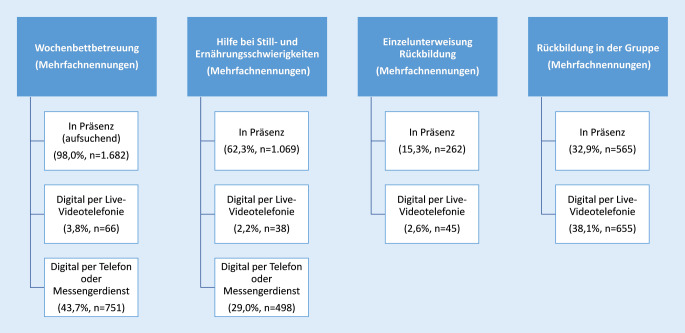

Die gesetzlichen Hebammenleistungen im Wochenbett wurden ebenfalls je nach Leistung unterschiedlich häufig in Präsenz oder digital in Anspruch genommen. Die „Wochenbettbetreuung“, die Untersuchungen von Mutter und Kind beinhaltet, wurde fast ausschließlich (98,0 %, *n* = 1682) in Präsenz wahrgenommen (Abb. 3). An der „Rückbildung in der Gruppe“ haben hingegen mehr als ein Drittel der Frauen digital per Live-Videotelefonie teilgenommen.

Private Hebammenleistungen in der Schwangerschaft (wie z. B. Schwangeren-Yoga oder Hypnobirthing) und nach der Geburt (wie z. B. Trageberatung oder Babymassage) hat jede 4. bis 5. Frau in Präsenz in Anspruch genommen, aber nur sehr wenige Frauen in digitaler Form.

### Bewertung der digitalen Angebote

**Figure 4:**
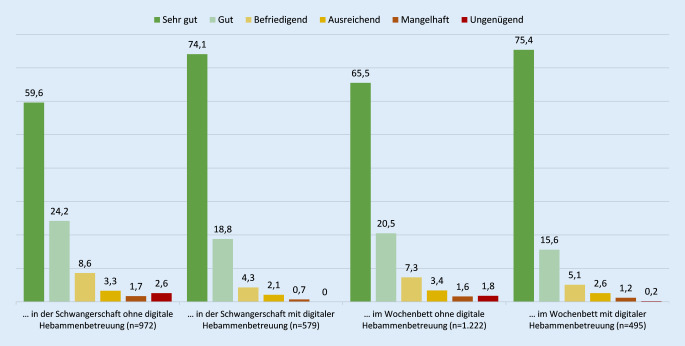

Mütter, die digitale Betreuung in Anspruch genommen haben, bewerteten diese sowohl in der Schwangerschaft (92,9 %, *n* = 538) als auch im Wochenbett (91,0 %, *n* = 450) fast durchweg positiv und insgesamt sogar etwas besser als Mütter, die ausschließlich in Präsenz betreut wurden (Abb. 4). Die Bewertung wurde mittels einer 6‑stufigen Skala (analog zu Schulnoten – sehr gut (=1), gut (=2), befriedigend (=3), ausreichend (=4), mangelhaft (=5), ungenügend (=6)) erhoben. Frauen, deren Betreuung ausschließlich in Präsenz stattgefunden hatte, haben diese durchschnittlich mit der Note 1,7 in der Schwangerschaft und mit 1,6 im Wochenbett bewertet. Frauen mit (zusätzlicher) digitaler Betreuung haben sowohl für die Betreuung in der Schwangerschaft als auch im Wochenbett durchschnittlich die Note 1,4 vergeben.

Als größte Chance wird die Möglichkeit der digitalen Betreuung trotz eigener Quarantäne (in der Schwangerschaft: 39,7 % (*n* = 230) und im Wochenbett: 32,3 % (*n* = 160)) wahrgenommen und auch der Wegfall des Fahrtweges sowie die damit einhergehende Zeitersparnis werden als große Chance betrachtet. Auffallend positiv ist zudem, dass Frauen die digital betreut wurden, zu 84,3 % in der Schwangerschaft und zu 90,1 % im Wochenbett keinerlei Herausforderungen im digitalen Betreuungsformat sahen.

Darüber hinaus gab mehr als jede 3. Mutter mit digitaler Betreuung an (34,2 %, *n* = 250), dass diese „genau richtig“ war. Dennoch wünschte sich jede 5. Mutter (18,1 %, *n* = 132) einen besseren Austausch mit anderen Kursteilnehmerinnen und jede 4. Mutter (24,8 %, *n* = 181) plädierte für mehr Interaktivität in den digitalen Kursen.

### Frauen mit Migrationshintergrund

Insgesamt haben 100 Frauen (5,5 %) mit Migrationshintergrund an der Befragung teilgenommen, somit sind die folgenden Ergebnisse nicht repräsentativ, werden aber trotzdem genannt, da Tendenzen aufgezeigt werden können. Die Altersstruktur ist ähnlich wie in der Gesamtstichprobe und es haben vor allem Frauen teilgenommen, die ihr erstes Kind zur Welt gebracht haben (45,0 %, *n* = 45). Diskrepanzen zwischen Frauen mit und ohne Migrationshintergrund bestehen hinsichtlich der Betreuung durch eine Hebamme, wie Tab. 2 zeigt. Die Betreuungsquote durch eine oder mehrere Hebammen in der Schwangerschaft (61,0 % vs. 79,2 %) und im Wochenbett war in der Gruppe der Frauen mit Migrationshintergrund etwas geringer als bei Frauen ohne Migrationshintergrund (78,0 % vs. 94,2 %). Jedoch wurden im Wochenbett 40,5 % (*n* = 32) der Frauen mit Migrationshintergrund digital betreut, im Gegensatz zu 28,3 % (*n* = 463) der Frauen ohne Migrationshintergrund (Tab. 2).

**Table 2:** 

		Frauen mit Migrationshintergrund (*n* = 100) (%)	Frauen ohne Migrationshintergrund (*n* = 1721) (%)
*Schwangerschaft*	Ich wurde während der Schwangerschaft von einer oder mehreren Hebamme(n) betreut	61,0	79,2
	Ich habe am Kursangebot einer oder mehrerer Hebamme(n) während der Schwangerschaft teilgenommen	30,0	55,4
	Ich wurde während der Schwangerschaft mit meinem jüngsten Kind digital von meiner Hebamme betreut	35,3	37,4
*Wochenbett*	Ich werde/wurde im Wochenbett von einer oder mehreren Hebamme(n) betreut	78,0	94,2
	Ich habe an einem Kursangebot einer oder mehrerer Hebamme(n) nach der Geburt teilgenommen oder nehme gerade an einem Kursangebot teil	41,0	56,3
	Ich wurde während des Wochenbetts mit meinem jüngsten Kind digital von meiner Hebamme betreut	40,5	28,3

Mehr als 80,0 % der befragten Frauen mit Hebammenbetreuung in der Schwangerschaft und im Wochenbett bewerteten diese positiv. Dennoch sehen Frauen mit Migrationshintergrund seltener Chancen in der digitalen Hebammenbetreuung in der Schwangerschaft (58,3 % *n* = 10 vs. 72,6 % *n* = 152). Anders ist es bei der digitalen Hebammenbetreuung im Wochenbett, diese bietet nach Ansicht von 75,0 % (*n* = 24) der Frauen mit Migrationshintergrund Chancen und für 71,7 % (*n* = 332) der Frauen ohne Migrationshintergrund. Die Herausforderungen digitaler Hebammenbetreuung bewerten beide Gruppen nahezu gleich.

## Diskussion

Freiberufliche Hebammen in Deutschland haben ihr Angebotsspektrum in kurzer Zeit verändert bzw. erweitert und bieten zusätzlich digitale Hebammenleistungen an [28]. Die Phase des Mutter- bzw. Elternwerdens ist eine zum Teil herausfordernde Lebensphase, die mit vielen Veränderungen einhergeht. Eine (kontinuierliche) Hebammenbetreuung in dieser Lebensphase unterstützt Frauen und ihre Familien physisch und psychisch. Insbesondere in Pandemiezeiten ist eine Unterstützung junger Familien vonnöten, hier spielt die Hebammenbetreuung eine wichtige Rolle. Digitale Angebote in der Schwangerschaft und im Wochenbett können dabei unterstützen und durch die effizientere Betreuungsmöglichkeit (Zeit- und Wegeersparnis) dazu beitragen, den Hebammenmangel zumindest etwas auszugleichen [26].

In der vorliegenden Studie haben 579 (37,3 %) der 1551 befragten Frauen, die in der Schwangerschaft von einer oder mehreren Hebammen betreut wurden, digitale Angebote in Anspruch genommen. Am häufigsten kam die digitale Live-Videotelefonie im Rahmen der „Geburtsvorbereitung in der Gruppe“ (27,0 %, *n* = 419) zum Einsatz. Das Vorgespräch in der Schwangerschaft wurde hingegen vorrangig in Präsenz geführt. Dies kann daran liegen, dass im Vorgespräch ein Kennenlernen der Frau und der Hebamme stattfindet und eine Vertrauensbasis geschaffen werden kann, die ein gutes Fundament für die weitere digitale Betreuung bietet.

Die Wochenbettbetreuung wurde fast ausschließlich (98,0 %, *n* = 1682) in Präsenz durchgeführt. An Rückbildungskursen nach der Geburt haben mehr als ein Drittel der Frauen digital per Live-Videotelefonie teilgenommen.

Zu bedenken ist, dass Frauen eventuell mehr und weitere digitale Hebammenleistungen in Anspruch genommen hätten, wären diese Angebote zugänglich gewesen. Die Ergebnisse der Hebammenbefragung deuten darauf hin, dass nicht alle Hebammen ihr Leistungsspektrum um digitale Angebote erweitert haben [26]. Insbesondere in der Wochenbettbetreuung müssen Untersuchungen an Mutter und Kind durchgeführt werden, die digital nur schwer bzw. nicht durchführbar sind [24–27].

Aus den Ergebnissen geht hervor, dass digitale Live-Videotelefonie eine Alternative zu Geburtsvorbereitungs- und Rückbildungskursen in Präsenz darstellt. Durch die Nutzung des digitalen Mediums wird das Infektionsrisiko minimiert und soziale Interaktion kann trotzdem stattfinden. Dennoch merkte jede 5. Mutter (18,1 %, *n* = 132) an, dass sie sich einen besseren Austausch mit anderen Kursteilnehmerinnen wünsche. Jede 4. Mutter (24,8 %, *n* = 181) möchte mehr Interaktivität in den digitalen Kursen. Der soziale Austausch der Schwangeren und neuen Mütter/Eltern untereinander ist wichtig und beugt Einsamkeit, Isolation und Überforderung vor. Inzwischen existieren Fortbildungsangebote für Hebammen, die den Umgang mit Videokonferenzen und deren interaktive Möglichkeiten vermitteln. Die vorliegende Befragung nimmt die Betreuung von Frauen zu Beginn der COVID-19-Pandemie im Jahr 2020 in den Blick. Heute – nach 2,5 Jahren – würden die Ergebnisse bzgl. des Angebotes ggf. anders ausfallen, da die Digitalisierung im Gesundheitsbereich weiter fortgeschritten ist.

Insgesamt zeigen die Ergebnisse, dass Frauen, die digitale Hebammenangebote genutzt haben, diese fast durchweg positiv bewertet haben. In der Schwangerschaft bewerteten 92,9 % (*n* = 538) und im Wochenbett 91,0 % (*n* = 450) der Mütter die erfahrene digitale Betreuung als positiv. Die wenigsten Frauen sehen Herausforderungen in der digitalen Betreuung, aber etliche Chancen. Da bisher keine vergleichbaren Befragungen von Frauen zur Nutzung und Bewertung von digitalen Hebammenleistungen existieren, ist ein Vergleich nicht möglich.

An der Befragung haben 100 Frauen (5,5 %) mit Migrationshintergrund teilgenommen, so dass diese Ergebnisse nur vorsichtig zu interpretieren sind. Insgesamt zeigt sich bei diesen Frauen eine niedrigere Betreuungsquote durch eine oder mehrere Hebammen in der Schwangerschaft und im Wochenbett. Es zeigte sich bereits in mehreren Erhebungen, dass der Zugang zur Hebammenbetreuung für Frauen mit Migrationshintergrund erschwert ist [2].

Dennoch wurden die befragten Frauen mit Migrationshintergrund im Wochenbett häufiger digital betreut als Frauen ohne Migrationshintergrund (40,5 % vs. 28,3 %). Die Mütter sehen seltener Chancen in der digitalen Hebammenbetreuung in der Schwangerschaft, aber im Wochenbett sehen 75,0 % (*n* = 24) Chancen in der digitalen Betreuung. Die Herausforderungen bewerten Frauen mit und ohne Migrationshintergrund nahezu gleich. Auch hier liegen keine vergleichbaren Studien vor, so dass diese Ergebnisse erst einmal nur Tendenzen aufzeigen können.

### Stärken und Limitationen der Studie

Bisher wurden unserer Kenntnis nach keine vergleichbaren Befragungen von Müttern im Kontext der digitalen Hebammenbetreuung während der COVID-19-Pandemie in Deutschland durchgeführt. Die erzielte Stichprobengröße von 1821 Frauen bei einer kurzen Studienlaufzeit ist positiv hervorzuheben. Die explorative Entwicklung der beiden Fragebögen (Frauen und Hebammen) erfolgte partizipativ und vielfältige Perspektiven und Professionen wurden in die Entwicklung miteinbezogen [29].

Die Stichprobe der befragten Mütter ist nicht repräsentativ für Frauen, die zwischen Mai und November 2020 in Deutschland ein Kind geboren haben. Insgesamt ist die Stichprobe recht homogen, die Frauen sind älter und gebildeter und haben zu einem geringeren Prozentsatz eine Migrationsgeschichte. Eine für schriftliche Befragungen typische Unterrepräsentanz bestimmter Gruppen von Frauen ist ersichtlich.

Der Rücklauf lag bei knapp 10 %. Möglicherweise liegt dies auch an Schwierigkeiten bei der Verwendung des QR-Codes im postalischen Anschreiben. Zudem muss bedacht werden, dass die angesprochenen Personen Mütter mit zum Teil noch jungen Babys sind, die wahrscheinlich sehr in die Care-Arbeit, also Tätigkeiten der Fürsorge, des Pflegens und Sich-Kümmerns, eingespannt sind.

Der verwendete Fragenbogen wurde explorativ entwickelt, da im Jahr 2020 in Deutschland sowie international noch keine validierten Instrumente zum Thema existierten [26, 30].

## Fazit

Die Ergebnisse liefern wichtige Hinweise bezüglich der Akzeptanz und Beurteilung der digitalen Hebammenbetreuung während der Schwangerschaft und im Wochenbett aus Sicht der Mütter. Dies ist bedeutend, da das Digitale-Versorgung-und-Pflege-Modernisierungs-Gesetz (DVPMG; [9]) seit Juni 2021 verstetigt wurde und daher Hinweise für Verbesserungen für die Verstetigung der digitalen Hebammenbetreuung genutzt werden können.

Weitere Forschung ist notwendig, um z. B. im Rahmen einer Längsschnittstudie die Auswirkungen der verstetigten digitalen Hebammenbetreuung zu untersuchen. Hierbei sind die Auswirkungen auf die erforderlichen Kompetenzen und Tätigkeiten der Hebammen sowie auf die Versorgungsqualität von Interesse. Darüber hinaus ist es wichtig, die Bedürfnisse und Bedarfe von Frauen hinsichtlich digitaler Hebammenbetreuung zu evaluieren, um passgenaue Angebote für die Betreuung vor und nach der Geburt machen zu können. Dabei wäre es förderlich, Frauen miteinzubeziehen, die sich in schwierigen sozialen Lagen befinden oder Belastungen ausgesetzt sind, da bekannt ist, dass insbesondere diese Gruppen von Frauen einen erschwerten Zugang zur Hebammenbetreuung haben, aber sehr von dieser profitieren könnten. Um noch mehr Evidenz zu schaffen, ist es wichtig, darüber hinaus Erkenntnisse dazu zu gewinnen, wo und wie der Einsatz von digitalen Betreuungsformen sinnvoll ist und welche Technologien geeignet sind und eine Verbesserung der Versorgung bewirken.

Die Chancen der Digitalisierung im Gesundheitssektor sollten genutzt werden und langfristig sollte eine sinnvolle Kombination aus digitalen Angeboten und Angeboten in Präsenz in der Hebammenversorgung umgesetzt werden.

### Infobox 1

Im Rahmen der Studie „Digitale Hebammenbetreuung im Kontext der Covid-19-Pandemie“ wurden freiberufliche Hebammen und Mütter nach der Geburt befragt. In diesem Beitrag werden die Ergebnisse der Mütterbefragung dargestellt. Den kompletten Abschlussbericht finden Sie hier: https://www.hs-gesundheit.de/fileadmin/user_upload/Forschung/Abschlussbericht_Digiheb_31.08.2021.pdf.

## Footnotes

1 Der Hebammenfragebogen wird in diesem Artikel größtenteils ausgeklammert. Die gesamten Ergebnisse der Studie finden Sie unter: https://www.hs-gesundheit.de/fileadmin/user_upload/Forschung/Abschlussbericht_Digiheb_31.08.2021.pdf.

2 Mother Hood e. V. ist eine Elterninitiative, die sich für sichere Geburten und die Rechte von Frauen und Familien einsetzt. Die Initiative vertritt die Interessen von Frauen und Familien gegenüber Hebammen, Ärzt*innen, der Politik und Wissenschaft (https://mother-hood.de/).

3 Kinderheldin ist eine interaktive Kursplattform für die Schwangerschaft und die Zeit mit dem Kind und bietet über 50 Online-Kurse an. Bei der BARMER Versicherte können die Hebammenberatung per Chat und Telefon jederzeit und so oft wie gewünscht kostenlos nutzen. Zudem erhalten Versicherte Zugang zum Kinderheldin-Stillkurs mit praktischen Tipps und wichtigen Informationen zur Stillzeit (https://kinderheldin.de/barmer#/code).
